# Sodium Fluorescein for Spinal Intradural Tumors

**DOI:** 10.3389/fonc.2020.618579

**Published:** 2021-01-28

**Authors:** Semih Kivanc Olguner, Ali Arslan, Vedat Açık, İsmail İstemen, Mehmet Can, Yurdal Gezercan, Ali İhsan Ökten

**Affiliations:** Department of Neurosurgery, Adana City Training and Research Hospital, Adana, Turkey

**Keywords:** guidance, surgical resection, spinal tumor, sodium fluorescein, intradural tumor

## Abstract

Technological innovations in spinal intradural tumor surgery simplify treatment. Surgical treatment of cranial benign and malignant pathologies under microscope with sodium (Na)-fluorescein guidance has often been reported, but few studies have focused on spinal intradural tumors. We aimed to investigate the usefulness of Na-fluorescein under yellow filter in intradural spinal tumor surgery by retrospectively reviewing cases involving intramedullary and extramedullary tumors operated under the guidance of Na-fluorescein. Forty-nine adult patients with a diagnosis of spinal intradural tumor operated under a yellow filter (560 nm) microscope using Na-fluorescein dye were included in the study. Demographic data, such as age and sex, neurological status, extent of tumor resection, histopathological diagnosis, Na-fluorescein staining pattern, and its usefulness during surgery were noted and statistically analyzed. Of all recruited patients, 26 women (53.1%) and 23 men (46.9%), were included for analysis. The age range of the patients was 18–64 years, with a mean age of 41.6 ± 13.9. An intradural intramedullary mass was found in 30.6% (n = 15) of the patients, and an intradural extramedullary mass in 69.4% (n: 34). While Na-fluorescein staining was homogeneous in all intradural extramedullary tumors, 73.3% (n: 11) of intradural intramedullary tumors were homogeneous, and 13.3% (n: 2) moderately heterogeneous. In the whole study group, the Na-fluorescein staining pattern was helpful in surgical resection in 47 cases (95.9%). While 34/34 (100%) found it helpful for extramedullary tumors, 13/15 (86.7%) did in intramedullary tumors, and for 2/15 (13.3%) it was not. In conclusion, Na-fluorescein helps in distinguishing tumor from healthy tissue in intradural extramedullary and intramedullary tumor surgery under a yellow filter microscope in most cases, thus providing convenient assistance to surgeons.

## Introduction

Spinal intradural tumors are rare central nervous system (CNS) diseases categorized as intramedullary and extramedullary according to their location. Treatment of these pathologies is surgical, and in recent years effective results have resulted in tumor surgery from the use of technological innovations. Intraoperative neuromonitorisation (IONM), magnetic resonance (MR) tractography, and ultrasonography (USG) have been used for safe and maximum surgical resection (1–10). Furthermore, surgery under the guidance of Sodium-fluorescein (Na-fluorescein) has earned a place in neurosurgery for tumor resection and tumor volume reduction. This technique, widely used in intracranial malignant and benign tumor surgery, provides an advantage to the surgical team by staining the tumor tissue in cases where the blood-brain barrier is disrupted (11–16). Similarly, in spinal intradural tumors, another pathology of the central nervous system, the blood-brain barrier is disrupted during neoplasia development. As with intracranial CNS tumors, the main goal is to perform maximal surgical tumor resection within safe limits in spinal intradural masses. Providing gross total resection (GTR) also positively affects the patient’s overall quality of life by affecting progression-free survival (PFS) and long-term neurological improvement (LTNI) (17, 18). While tumor margins are clearly monitored in extramedullary tumors, it may not always be possible to find these borders in intramedullary tumors. The most important factor determining the gross total resection is the perioperative tumor cleavage plan (17). For this reason, we assumed that surgery under a yellow filter in the presence of Na-fluorescein might be effective in determining the tumor cleavage plan, especially for spinal intradural tumors. The aim of this study was, therefore, to examine the staining pattern of Na-fluorescein in intradural intramedullary and extramedullary tumors and investigate its suitability for surgery. To our knowledge, only two studies evaluated spinal intradural tumors under yellow filter microscope in the presence of Na-fluorescein (19, 20). Considering the number of published articles evaluating the use of Na-fluorescein in intracranial CNS tumors, this number is very low, pointing to a gap in knowledge we aimed to fill with the data gathered in this study.

## Material and Methods

### Patients and Data

This study was carried out in accordance with the principles of the Declaration of Helsinki and approved by the Ethics Committee of Adana City Training and Research Hospital. Informed consent was obtained from all patients. Inclusion criteria for the study were adult patients operated under PENTERO 900 (Carl Zeiss, Meditec, Oberkochen, Germany) and a microscope yellow filter (560 nm) using Na-fluorescein dye (5 mg/kg) with a diagnosis of spinal intradural tumor between May 2017 and May 2019. The exclusion criteria for the study were as follows: patients <18 years of age who were not given Na-fluorescein, with known hepatic and renal insufficiency, or those with a history of excessive hypersensitivity-allergic reactions. Besides demographic data such as age and sex of the patients, extent of tumor resection, histopathological diagnosis, Na-fluorescein staining pattern, and neurological status were noted and analyzed. For neurological evaluation, we employed the classification by McCormick et al. (21).

### Surgery

In all cases, 5 mg/kg Na-fluorescein was intravenously administered during anesthesia induction and imaging was performed under a microscope with a yellow 560 filter. All surgeries were performed by the same team with continuous intraoperative neuro-monitorization (somatosensory evoked potentials, motor evoked potentials, and electromyography). Level determination for tumor localization was performed under lateral view with C-arm fluoroscopy with patients placed in the prone position on the operating table. Following skin incision, the paraspinal muscles were dissected subperiosteally. After hemilaminectomy or laminectomy, the dura was opened, the yellow filter activated, and, if there was Na-fluorescein involvement, extramedullary tumors immediately observed. In intramedullary tumors, after myelotomy, the tumor was observed according to Na-fluorescein involvement, and the tumor-spinal cord tissue demarcation line searched. Tumor excision was performed using the classical microsurgical technique. Additional instrumentation was applied after duraplasty, laminoplasty, or according to the surgical team’s preference. The staining pattern was defined by consensus of surgeons performing the surgery. Similar to the classification used in the work by Millesi et al., they were divided into three groups, namely, dense homogeneous, moderate heterogeneous and none (22). If the tumor tissue was in vivid homogenous yellow color, it was determined as “dense homogeneous”, if lightly yellow in appearance “moderate heterogeneous” and if there was no staining determined as “none”.

### Radiological Assessment

Preoperative and postoperative (first 48 h) non-contrast and contrast-enhanced magnetic resonance imaging was performed in all patients, and the amount of resection noted. The basic algorithm preferred by Klekamp et al. was used to define the amount of resection (23). Cases whose tumor tissue was completely excised and no remnant was confirmed by postoperative MRI were called gross total resection (GTR), those with little tumor residue subtotal resection (STR), and those with >50% residual postoperative resection were called partial resection (PR).

### Statistical Analysis

Statistical evaluation was performed using the Statistical Package for Social Sciences (SPSS) for Windows 20 (IBM SPSS Inc., Chicago, IL). Normal data distribution was evaluated using the Kolmogorov-Smirnov test. Normally distributed numerical variables are shown as mean ± standard deviation, while numerical variables not showing normal distribution are shown as median (minimum, maximum). Categorical variables are expressed as numbers and percentages. Chi-square and Fisher’s exact chi-square tests were used for comparison of categorical data. Student’s t-test was used to compare numerical variables showing normal distribution between the two groups. For comparing the postoperative and preoperative period, a repeated mixed model analysis was used. In statistical analysis, a p <0.05 (*) is considered significant.

## Results

Between 2017 and 2019, 49 patients, comprising 26 women (53.1%) and 23 men (46.9%), with a diagnosis of spinal intradural tumor and operated using a yellow filter in the presence of Na-fluorescein, were included in the study. The age range of the patients was 18–64 years, with a mean age of 41.6 ± 13.9. An intradural intramedullary mass was found in 30.6% (n: 15) of patients, and intradural extramedullary mass in 69.4% (n = 34). According to anatomical tumor localizations, 36.7% (n = 18) were found to be cervical, 36.7% (n = 18) thoracic, and 26.6% (n: 13) lumbar. The preoperative McCormick scale was 1.5 ± 0.5 (min: 1; max: 3) and mostly 1.

Age and gender distribution did not differ between patients with intradural intramedullary and intradural extramedullary masses. While Na-fluorescein fluorescence was dense homogeneous in all intradural extramedullary tumors, 73.3% (n: 11) of intradural intramedullary tumors were dense homogeneous, and 13.3% (n: 2) were moderately heterogeneous. All masses with intradural extramedullary location were removed by gross total resection.

Subtotal resection was performed in 20% (n: 3) of intradural intramedullary masses, whereas gross total resection was performed in the others (**Table 1**). In the whole study group, the Na-fluorescein staining pattern was found helpful for surgical resection in 47 cases (95.9%). While in 34/34 (100%) cases it was helpful for all extramedullary tumors, 13/15 (86.7%) were helpful in intramedullary tumors and 2/15 (13.3%) were not helpful at all. In tumors with contrast enhancement on preoperative MRI, Na-fluorescein staining was also observed.

**Table 1:** Demographic and clinical features.

Variables	Total n = 49	Intramedullary n = 15	Extramedullary n = 34	p
Gender, n(%)				
Female	26(53.1)	8(53.3)	18(52.9)	0.999
Male	23(46.9)	7(46.7)	16(47.1)	
Age, years	41.6 ± 13.9	39.4 ± 13.9	41.1 ± 14.5	0.700
Na-Fl fluorescence, n(%)				
**Dense homogeneous** (meningioma, schwannoma, ependymoma, non-small cell lung cancer, hemangiopericytoma, ganglioneuroma)	45(91.8)	11(73.3)	34(100.0)	0.006*
**Moderate heterogeneous** (oligodendroglioma, pilocytic astrocytoma)	2(4.1)	2(13.3)	–	
**None** (dermoid and epidermoid tumors)	2(4.1)	2(13.3)	–	
Helpful, n(%)				
Helpful	47(95.9)	13(86.7)	34(100.0)	0.164
Not helpful	2(4.1)	2(13.3)	–	
Extent of resection, n(%)				
GTR	46(93.9)	12(80)	34(100.0)	0.164
STR	3(6.1)	3(20)	–	

Numerical variables were expressed as mean ± standard deviation.

Categorical variables were expressed as numbers and percentages.

*p < 0.05 shows statistical significance.

Localization and histopathological distributions in patients with intradural intramedullary and intradural extramedullary masses are shown in **Table 2**. Localization did not significantly differ between groups. Further, there was no allergic hypersensitivity reaction or any complication due to the use of Na-fluorescein.

**Table 2:** Localization and Histopathological distribution.

Variables	Total n = 49	Intramedullary n = 15	Extramedullary n = 34	p
Localization, n(%)				
Cervical	18(36,7)	8(53,3)	10(29,4)	0,333
Thoracic	18(36,7)	4(26,7)	14(41,2)	
Lumbar	13(26,6)	3(20,0)	10(29,4)	
Histopathology				
Dermoid tm	1(2,0)	1(6,7)	–	< 0,001*
Ependymoma WHO grade 2	10(20,4)	10(66,7)	–	
Epidermoid	1(2,0)	1(6,7)	–	
Ganglioneuroma	1(2,0)	–	1(2,9)	
Meningioma psammomatous grade 1	2(4,0)	–	2(5,8)	
Meningioma transitional grade 1	5(10,2)	–	5(14,8)	
Meningioma meningothelial grade 1	5(10,2)	–	5(14,8)	
Metastasis	1(2,0)	1(6,7)	–	
Mesenchymal non-meningothelial tumor, Hemangiopericytoma WHO grade 1	1(2,0)	–	1(2,9)	
Myxopapillary ependymoma grade 1	1(2,0)	–	1(2,9)	
Oligodendroglioma	1(2,0)	1(6,7)	–	
Pilocytic astrocytoma WHO grade 1	1(2,0)	1(6,7)	–	
Schwannoma	19(38,8)	–	19(55,9)	

Categorical variables were expressed as numbers and percentages.

*p < 0.05 shows statistical significance.

**Figure 1:**
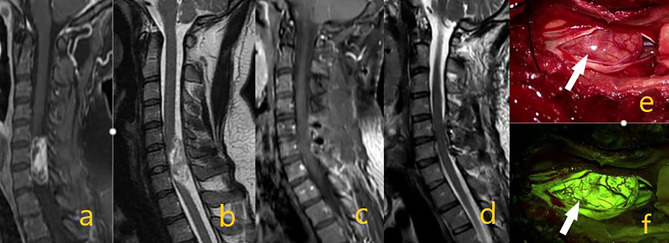
Schwannoma at the C6-7 level in preoperative contrast-enhanced T1 and T2 sequences on images **(A, B)**. On postoperative **(C, D)** images, the mass was totally removed. In intraoperative microscope image **(E)** white arrow shows the mass, while image **(F)** shows tumor homogeneous staining pattern with activated yellow filter.

Preoperative and postoperative McCormick scale scores were significantly higher in the intradural intramedullary group than in the intradural extramedullary group (**Table 3**). The postoperative McCormick scale was significantly increased in all patients compared to the preoperative period (1.5 ± 0.5 vs. 1.8 ± 0.6; p = 0.001). The proportion of patients with preoperative McCormick scale 1 decreased from 67.3 to 55.1% postoperatively, that of patients with McCormick scale 2 increased from 26.5 to 30.6%, as did that with McCormick scale 3 from 6.1 to 12.2% (**Table 4**). While the McCormick scale increased significantly in the postoperative period in the intradural intramedullary group compared to the preoperative period, there was no significant difference in the McCormick scale in the intradural extramedullary group (**Table 4**) (**Figure 1**).

**Table 3:** McCormick scale distributions.

McCormick scale	Total n = 49	Intramedullary n = 15	Extramedullary n = 34	p
Preoperative	1,5 ± 0,5	2,0 ± 0,7	1,1 ± 0,3	<0,001*
1	33(67,3)	3(20,0)	30(88,2)	<0,001*
2	13(26,5)	9(60,0)	4(11,8)	
3	3(6,1)	3(20,0)	–	
Postoperative	1,8 ± 0,6	2,5 ± 0,6	1,2 ± 0,4	<0,001*
1	27(55,1)	–	27(79,4)	<0,001*
2	15(30,6)	8(53,3)	7(20,6)	
3	6(12,2)	6(40,0)	–	
4	1(2,0)	1(6,7)	–	

Numerical variables were expressed as mean ± standard deviation.

Categorical variables were expressed as numbers and percentages.

*p < 0.05 shows statistical significance.

**Table 4:** Changes in McCormick scale measurements following the treatment.

Group	McCormick scale	Preoperative	Postoperative	p	p_d_
Total population	Scale	1,5 ± 0,5	1,8 ± 0,6	0,001*	
	1, n(%)	33(67,3)	27(55,1)	0,001*	–
	2, n(%)	13(26,5)	15(30,6)		
	3, n(%)	3(6,1)	6(12,2)		
	4, n(%)	–	1(2,0)		
Intra medullary	Scale	2,0 ± 0,7	2,5 ± 0,6	0,001*	< 0,001*
	1, n(%)	3(20,0)	–	0,005*	
	2, n(%)	9(60,0)	8(53,3)		
	3, n(%)	3(20,0)	6(40,0)		
	4, n(%)	–	1(6,7)		
Extra medullary	Scale	1,1 ± 0,3	1,2 ± 0,4	0,105	
	1, n(%)	30(88,2)	27(79,4)	0,083	
	2, n(%)	4(11,8)	7(20,6)		
	3, n(%)	–	–		
	4, n(%)	–	–		

Numerical variables were expressed as mean ± standard deviation.

Categorical variables were expressed as numbers and percentages.

*p < 0.05 shows statistical significance.

pd: Difference between groups of McCormick scale changes after treatment.

## Discussion

This study was conducted to investigate the viability of Na-fluorescein under a yellow filter in intradural spinal tumor surgery. The clinical process was evaluated by examining the tumor staining pattern in tumors with various histopathologies. Na-fluorescein has been used in ophthalmological angiography since 1961 (24). Known as a reliable agent, Na-fluorescein has been used under the YELLOW 560 filter in many pediatric and adult cranial neurooncological pathologies (15, 25–29).

In the literature, there are many studies examining spinal cord intradural tumors with 5-aminolevulinic acid (5-ALA) (30–33), but few on Na-fluorescein. In a comprehensive 5-ALA study, 5-ALA was positive in spinal intramedullary gliomas and the vast majority of intradural meningiomas, and proven to be a useful technique, especially in intramedullary gliomas (33). In the study by Millesi et al., a clinical series including 55 cases was published and 5-ALA evaluated as beneficial in intramedullary tumors (22). In our study, staining accompanied by Na-fluorescein was detected in all extramedullary tumors (34/34) and different degrees of staining in the majority of intramedullary tumors (13/15), and it was found that it helped surgery in 47 of 49 cases.

**Figure 2:**
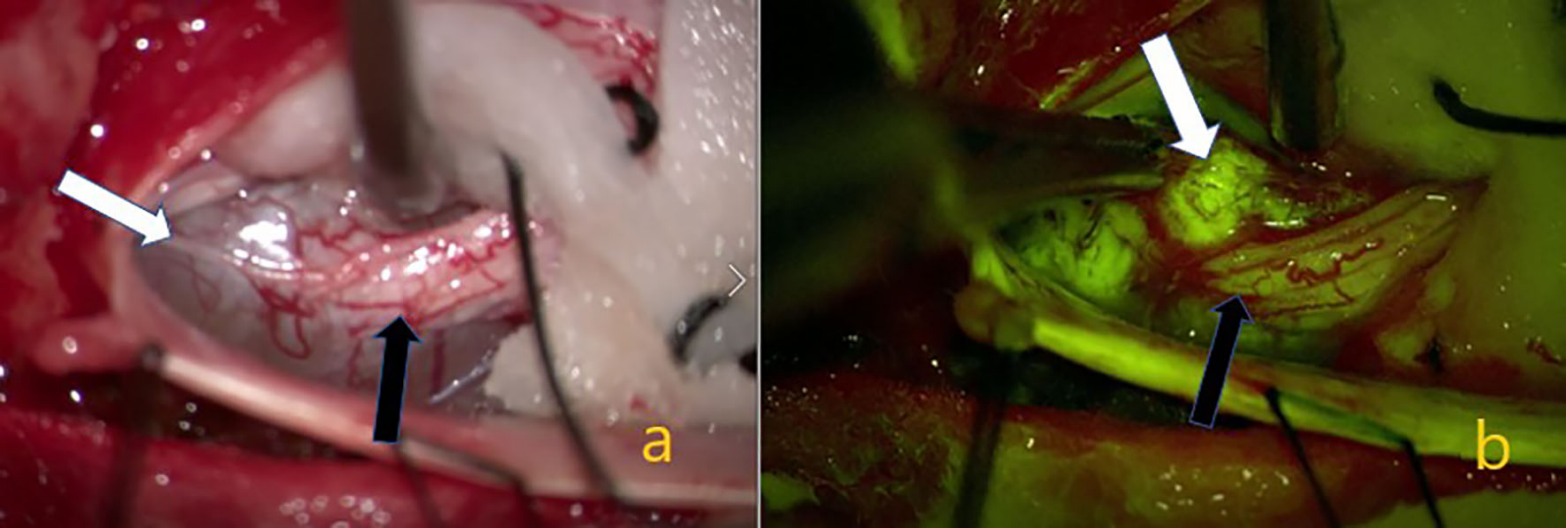
Schwannom located in the lumbar 5. **(A)** White arrow showing the tumor on the intraoperative microscope image. Black arrow indicating the nerve root from which the tumor originated. In **(B)** the yellow filter is activated, the white arrow indicating homogeneous staining of the tumor tissue, and the black arrow indicating weaker staining of the nerve root.

**Figure 3:**
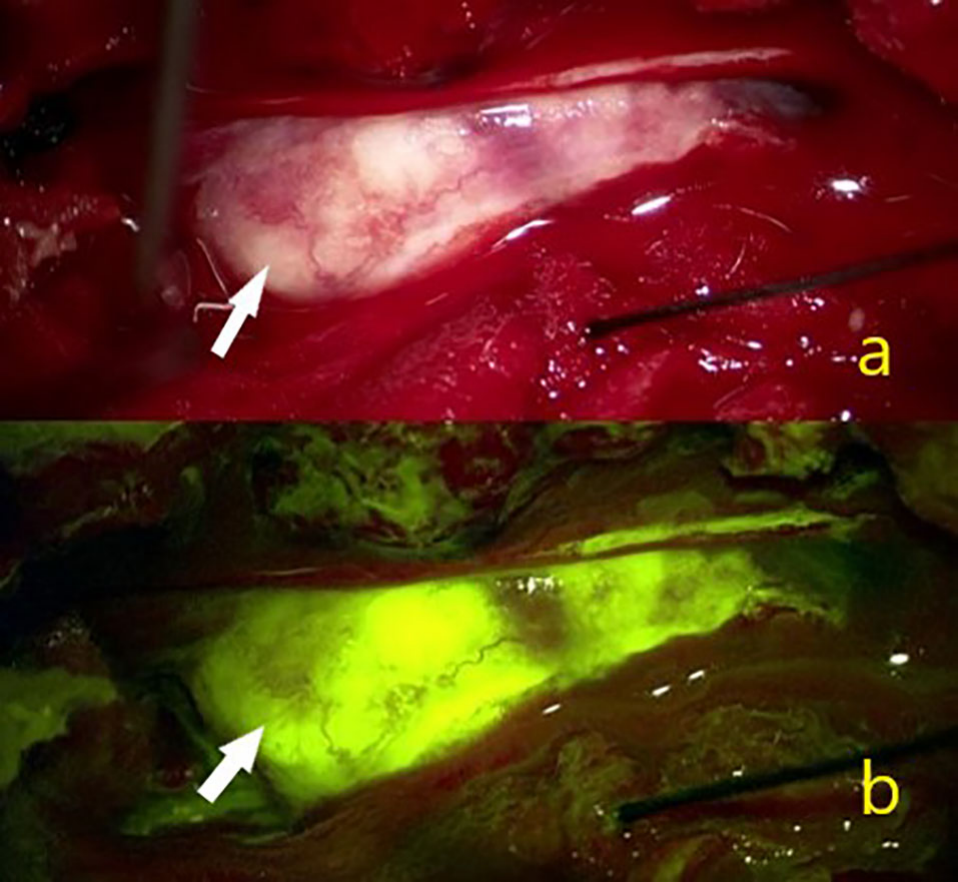
In a 42-year-old female patient **(A)** White arrow showing the intraoperative view of a T11 localized psammomatous meningioma. **(B)** White arrow indicating intense homogeneous Na-fl uptake.

According to our data, homogeneous Na-fluorescein involvement was detected in all extramedullary tumors (19 schwannomas, 12 meningiomas WHO grade 1, hemangiopericytoma WHO grade 1, ganglioneuroma and myxopapillary epandymoma WHO grade 1). In a prospective study in which Falco et al. evaluated cranial and spinal tumors in the presence of Na-fluorescein, it was reported that two cases of lumbar schwannoma were well stained (20). However, the authors thought that the use of Na-fluorescein in neurinomas was not necessary in surgical treatment because they were easily recognizable tumors, even if stained with Na-fluorescein in homogeneous intensity, and they failed to distinguish non-tumor nerve fibers. In our study, 19 Schwannomas WHO grade 1 were evaluated and, unlike the above view, an effective tumor-non-tumor nerve and fiber distinction could be made in every one of them (**Figures 1**, **2**). The reason for this divergence between the two studies may be that the Schwann cells which conform the tumor form different tumor subtypes. Generally referred to as Schwannoma WHO grade 1, these tumors have many histopathological subtypes (cellular, melanotic, neurofibroma/schwannoma hybrid tumors) (34). Another reason may be the difference in enhancement patterns on preoperative MRI. Spinal schwannomas have been reported to show different types of enhancement patterns (35, 36). Previous studies have reported that preoperative contrast enhancement is due to disruption of the blood brain barrier and contrast enhancement is correlated with Na-fluorescein involvement (27, 37, 38). Considering the above, we think that the use of Na-fluorescein in spinal Schwannomas is beneficial for tumor resection. In the future, we aim to gather more information on this subject. In our study, in which 12 WHO grade 1 meningiomas were evaluated, there were psammomatous, transitional, and meningothelial subtypes. In all of them, intense homogeneous staining and effective tumor-normal tissue separation were detected (**Figure 3**). All meningiomas were grossly excised. Simpson’s classification was used for resection classification of meningiomas, and a Simpson 2 resection was performed for all 12 meningiomas. In the literature, we did not find a single study using a yellow filter in the presence of Na-fluorescein in spinal meningiomas. However, it is known that Na-fluorescein has an intense staining pattern and there are high GTR rates in cranial meningiomas (20, 25). In their study, in which 30 patients were evaluated, Akçakaya et al. reported that surgery under YELLOW-560 filter, accompanied by Na-fluorescein, has an increasing effect on safety and extent of resection. In this context, we think that Na-fluorescein is useful both in preventing vascular injuries and distinguishing between tumor and healthy tissue in meningiomas with dense vascularity.

Gross total resection was performed for all extramedullary tumors (34/34). Except for schwannomas and meningiomas, myxopapillary ependymoma grade 1, hemangiopericytoma, and ganglioneuroma WHO grade 1 are rare tumors. In our study, these tumors had an intense homogeneous enhancement pattern on preoperative MRI. Under the yellow filter microscope image, an intense Na-fluorescein uptake was detected and the distinction between tumor and spinal cord tissue could be easily reached.

**Figure 4:**
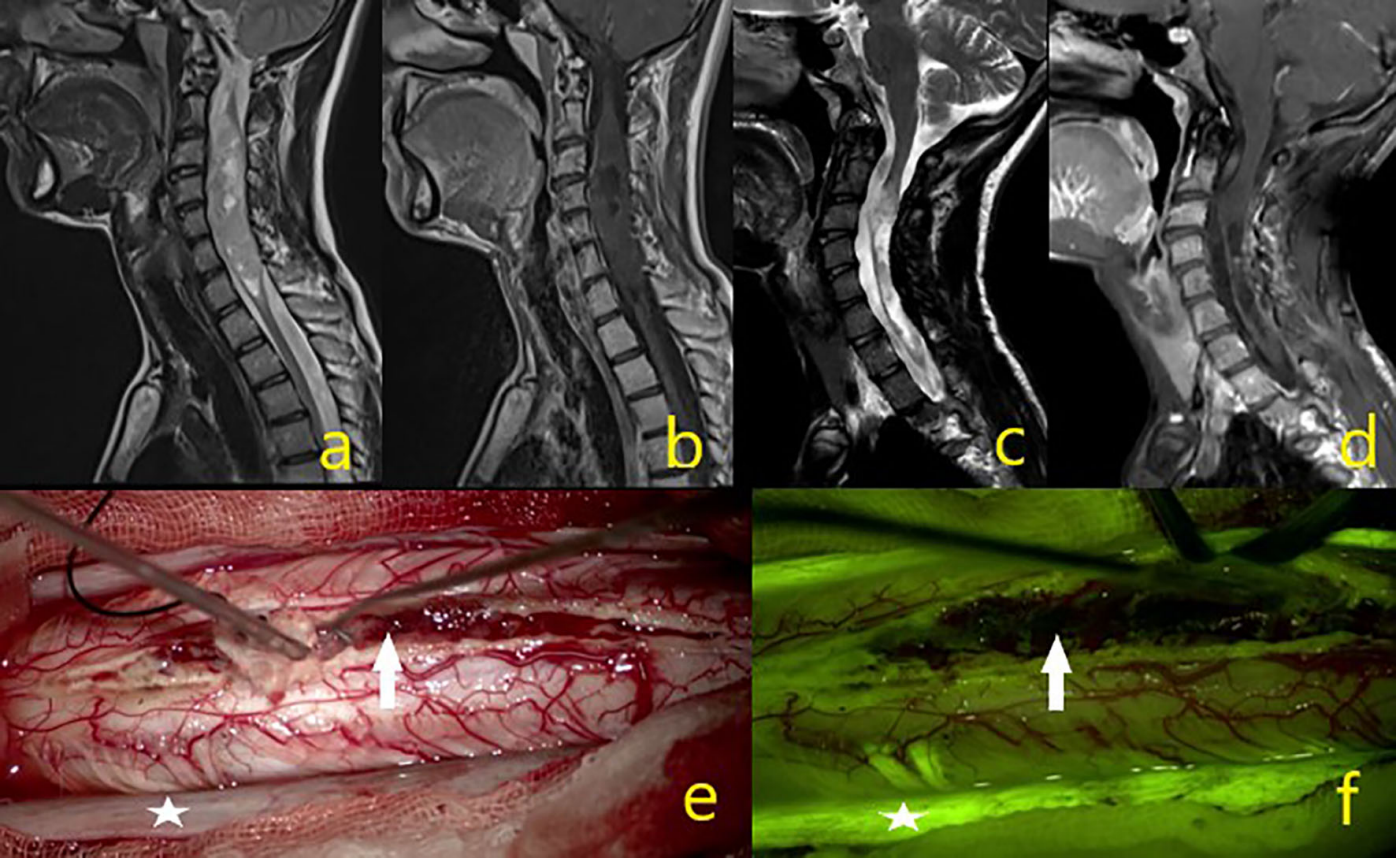
A 23-year-old male patient operated for oligodendroglioma WHO grade 2, but where subtotal excision could not be achieved due to intraoperative IONM signal loss. **(A–D)** Tumor MR sequence images. **(E)** In the intraoperative microscope image, the asterisk is the dura and the white arrow indicates the mass. **(F)** Under the yellow filter, the asterisk shows the dura staining pattern, and the white arrow a moderate heterogeneous stained mass image.

**Figure 5:**
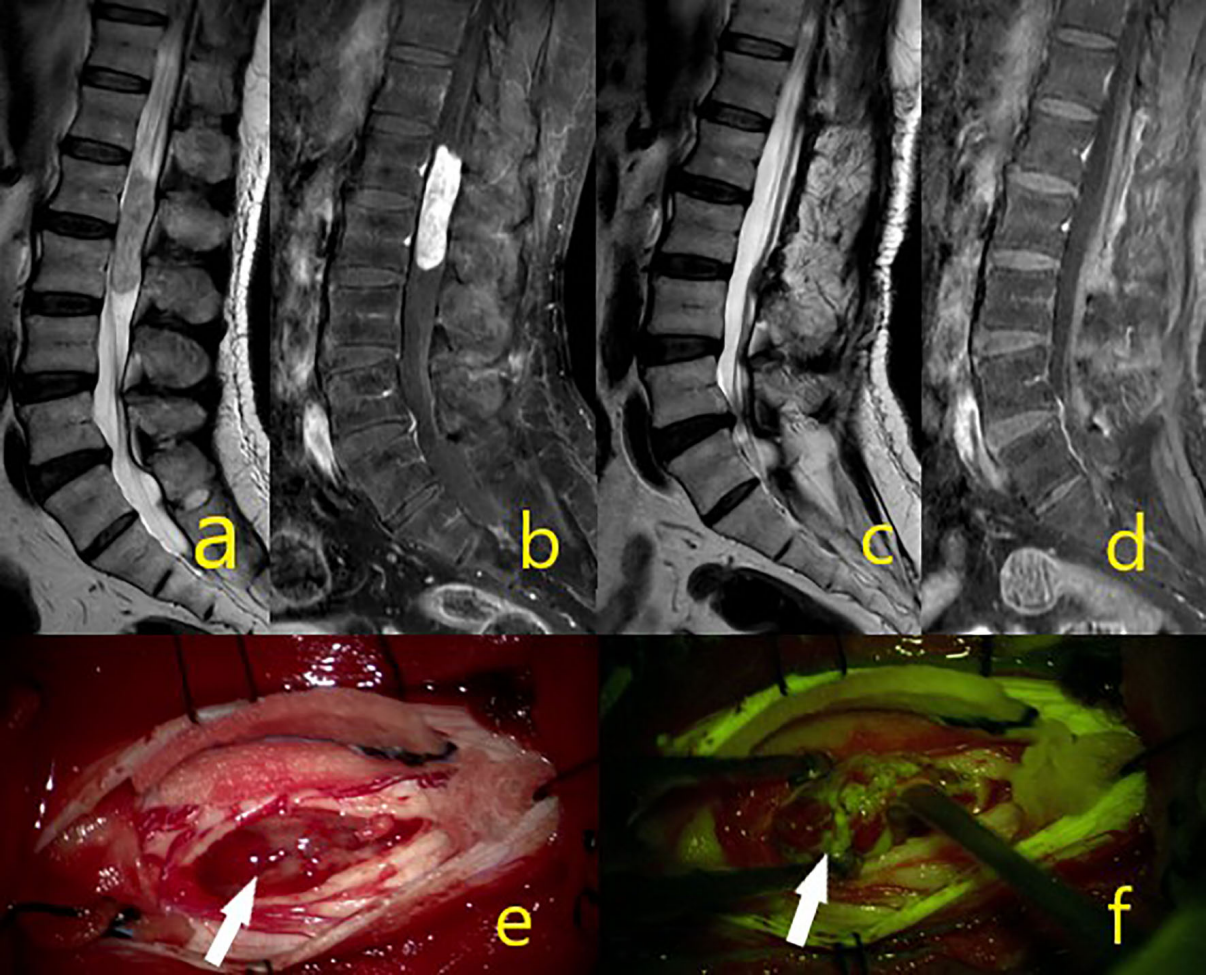
A 44-year-old male patient was operated from epandymoma WHO grade 2 and gross total excision achieved. **(A–D)** Tumor MR sequence images. **(E)** White arrow showing the mass in the intraoperative microscope image**. (F)** White arrow under yellow filter showing the dense homogenous stained mass image.

In intramedullary tumors, Na-fluorescein involvement was not detected in two patients (dermoid and epidermoid tumors). Moderate heterogeneous staining was detected in two patients (oligodendroglioma WHO grade 2 and pilocytic astrocytoma WHO grade 1), and the remaining 11 intramedullary tumors (10 ependymomas WHO grade 2, 1 non-small cell lung cancer) were detected with dense homogeneous staining, and the tumor demarcation line found. Among the intramedullary tumors, in a patient with oligodendroglioma WHO grade 2, although the tumor demarcation line was followed, surgery was terminated early due to signal loss in intraoperative neuro-monitorization and subtotal resection performed (**Figure 4**). In two patients diagnosed with ependymoma WHO grade 2, the surgery was terminated by performing subtotal resection due to early motor-evoked potential signal loss, but GTR was provided in all other intramedullary ependymomas. Studies have reported that ependymomas are well stained with 5-ALA and Na-fluorescein, which is useful in determining the tumor cleavage plan by distinguishing the tumor from healthy spinal tissue cord (19, 22, 30). Acerbi et al. presented their first experience of intramedullary tumor surgery under the YELLOW 560 filter with Na-fluorescein (19), wherein a total of 11 patients with various histopathologies were evaluated, all ependymomas (5/5) were homogeneously stained bright and subjected to gross total resection. In our study, 10 ependymomas were intensely stained in WHO grade 2 and GTR applied to eight of them (8/10) (80%) (**Figure 5**). STR had to be applied in two cases due to early signal loss in neuro-monitorization. Klekamp et al., presenting a huge series of 100 patients in intramedullary ependymoma surgery, stated that GTR ratio was 86.3% (23). The GTR rate was reported lower, namely, as 69 and 80% in two other important series (39, 40). The reason for not achieving GTR could be the absence of a tumor cleavage plan in ependymomas, loss of neurophysiological signal during surgery, and overlooking residual tumor tissue under white light (22, 30). In this study, the staining pattern and GTR rate obtained in ependymomas are satisfactory considering the above, thus, we consider Na-fluorescein useful in the surgical treatment of ependymomas with a yellow filter.

This study has some limitations. Performing a volumetric analysis, especially when reporting extent of resection results, could have yielded more objective results in terms of comparing preoperative and postoperative values. However, the high GTR rate in the study partly compensates for this disadvantage. Furthermore, the retrospective design and the limited number of cases in some of the anatomopathological diagnoses is a disadvantage. The strength of our study is that it is the first to report the results of surgical procedures using a yellow filter microscope to detect Na-fluorescein in a large case series consisting of 49 patients with spinal cord tumors.

In conclusion, an Na-fluorescein staining pattern under the yellow 560 filter in parallel with preoperative MR enhancement could be observed in both extramedullary and intramedullary spinal cord tumors. During surgery of both extramedullary and intramedullary tumors, it facilitates the distinction between intraoperative tumors and healthy tissue. Given the scarcity of research on this topic and its safety, its use in intradural spinal cord tumors will shed light on future studies.

## Data Availability Statement

The original contributions presented in the study are included in the article/supplementary materials. Further inquiries can be directed to the corresponding author.

## Ethics Statement

The studies involving human participants were reviewed and approved by Ethics Committee of Adana City Training and Research Hospital. The patients/participants provided their written informed consent to participate in this study.

## Author Contributions

YG and AÖ constructed the hypothesis. SO, AA, VA, and İİ made data collection and took responsibility in data management and reporting analysis. SO, AA, VA, İİ, and MC made literature review and wrote manuscript. YG and AÖ critically reviewed the article intellectually. All authors contributed to the article and approved the submitted version.

## Conflict of Interest

The authors declare that the research was conducted in the absence of any commercial or financial relationships that could be construed as a potential conflict of interest.
